# Patterns of surgical complications after delayed fixation of peripartum pubic symphysis rupture: a report of 5 cases

**DOI:** 10.1186/s13037-023-00381-w

**Published:** 2023-12-07

**Authors:** Grzegorz Doroszewski, Jan Wasielewski, Paweł Bartosz, Adam Caban, Anna Scholz, Jerzy Białecki

**Affiliations:** 1 Centre of Postgraduate Medical Education, Pelvic Injury and Pathology Department, Konarskiego 13, 05-400 Otwock, Poland; 2 Centre of Postgraduate Medical Education, 1st Department of Obstetrics and Gynecology, Żelazna 90, Warsaw, 01-004 Poland

**Keywords:** Symphysis disruption, Symphysis separation, Childbirth, Peripartum, Pelvic girdle pain, Pubic symphysis diastasis, Pregnancy complications, Postoperative complications

## Abstract

**Background:**

The disruption of the pubic symphysis during the peripartum period is a rare injury to the pelvic ring. In most cases, conservative treatment is successful. Nonetheless, there are cases where surgical intervention is necessary. We analyzed five surgical cases treated in our department and performed a literature review.

**Case presentations:**

Five women, ranging in age from 25 to 38, who experienced peripartum symphysis rupture were primarily treated with a conservative approach. Patients who did not show improvement and met certain criteria, such as experiencing pain starting from childbirth, having a separation in the pubic bone of more than 10 mm, and/or having a vertical instability greater than 5 mm, were recommended to undergo surgery. The average length of time between childbirth and surgery was 5.6 months, ranging from 1 to 14 months. One patient was treated with an external fixator, another patient received a combination of an external fixator and an anterior plate, and three patients were treated with anterior plates. In four cases, we observed a failure in fixation and a partial or complete loss of reduction*.* The plate and screws were removed in one case, and in three cases, revision surgery was performed. One case involved using a larger plate, while the other used 90-90 plating, known as “box plate fixation.” The mean follow-up was 7.4 years. Two cases had good results, and two had excellent results on the Lindahl scale.

**Conclusion:**

For patients with peripartum pubic symphyseal dislocation, our case series and literature review demonstrated that early reduction and fixation correlate with improved clinical outcomes and lower implant failure. For patients with subacute/chronic injuries, there was a higher incidence of implant failure. Orthogonal plate fixation and/or pubic symphysiodesis was associated with improved clinical outcomes.

**Supplementary Information:**

The online version contains supplementary material available at 10.1186/s13037-023-00381-w.

## Background

Based on computerized tomography (CT) investigations of female pelvises, the average width at the central part of the pubic symphysis is 4.60 ± 1.21 mm [1]. The physiological phenomenon of enhanced laxity in maternal pelvic ligaments is driven by relaxin [2]. In typical physiological circumstances, pregnancy induces a widening of the pubic symphysis by 1-3 mm, accompanied by the relaxation of ligaments in the iliosacral joints [2, 3]. However, a gap exceeding 10 mm in the pubic symphysis is deemed pathological [3].

Rapid fetal progression through the birth canal can potentially compromise the ligaments of the pubic symphysis, consequently affecting the anterior and posterior ligaments of the iliosacral joint [3, 4]. Biomechanically, this scenario is akin to the “open book” pelvic ring injury resulting from a high-energy force. It can be categorized as either an anteroposterior compression (APC) grade II or III, following the classification of pelvic ring injuries by Young and Burgess [5], or as Type B (partially unstable, rotationally unstable, vertically stable) or Type C (completely unstable) according to Tile [6].

Evidence-based risk factors for symphyseal separation include the rapid descent of the fetal head, a short second stage of labor, multiple pregnancies, multiparity, and spontaneous vaginal delivery [3].

In the majority of cases, peripartum pubic rupture can be successfully managed without surgical intervention [3, 7–10]. However, when the diastasis of the public symphysis exceeds 25 mm, conservative treatment may prove ineffective, and moderate to poor outcomes can be anticipated [4, 11]. Consequently, surgical intervention becomes necessary [3, 4, 8, 10].

Various surgical approaches have been described for this condition, including closed reduction (CREF), open reduction, and internal fixation (ORIF) of the pubic symphysis with or without iliosacral joint fixation, as well as a fusion of the pubic symphysis and iliosacral joint. Presently, the most prevalent method for fixing the pubic symphysis involves using an anterior plate [3, 8, 10].

The failure rate for anterior fixation in traumatic symphyseal rupture varies between 11 and 31%. Additionally, there is a risk ranging from 7 to 24% of losing the reduction achieved, with a 5 to 9% chance of requiring revision surgery. These rates are contingent on the severity of the injury and the specific fixation method employed. Furthermore, there is a suggestion [9] that delaying surgical intervention for peripartum symphysis rupture could potentially have an adverse effect on treatment efficacy.

Due to the low number of cases, there are no clear recommendations for surgical treatment methods. This paper aims to 1) analyze our experience with surgically treated peripartum pubic symphyseal dislocations, 2) compare our outcomes to the available literature, and 3) propose a treatment algorithm. We hypothesize that delaying surgical intervention has a negative effect on the outcomes of surgical treatment.

## Case reports

### Case 1

**Fig. 1:**
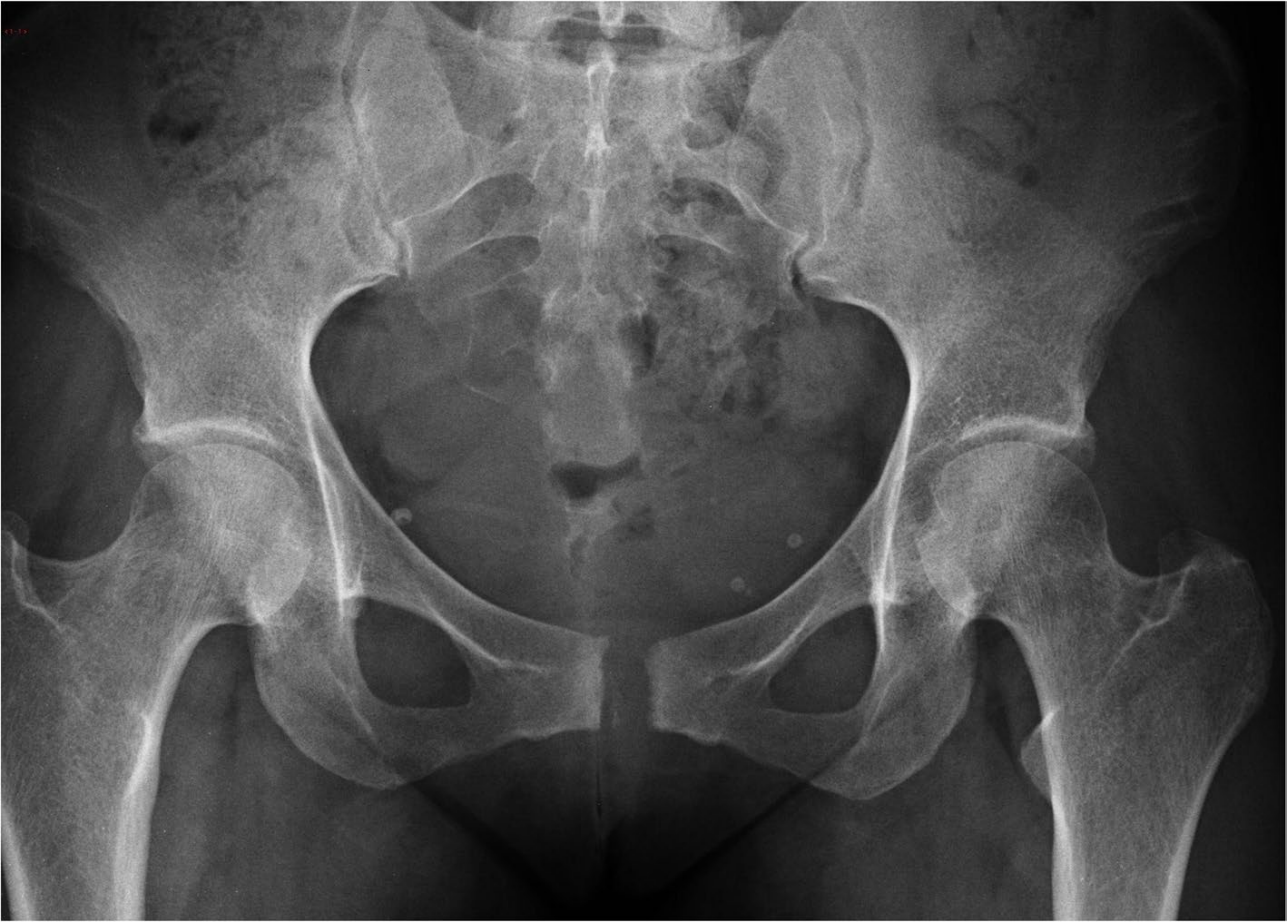
Case 1. A 34-year-old woman was treated with an external fixator with excellent results. A radiograph taken 1 year postoperatively showed a 12 mm diastasis

A 34-year-old woman (gravida I, para I) was admitted to the department 26 days after a natural vaginal delivery. The newborn weighed 4200 g and measured 54 cm, with the added complication of a clavicle fracture. The patient reported experiencing pain during labor localized in the region of the pubic symphysis and the right iliosacral joint. Initially, conservative treatment with a belt was attempted, but the patient remained unable to ambulate. The anteroposterior (AP) radiograph revealed a pubic symphysis rupture with a 70 mm separation width and a 7 mm vertical displacement. The right iliosacral joint also appeared widened, classifying it as a pelvic APC III injury. Closed reduction and external fixation were performed, and the patient was discharged home on postoperative day 7, using a walker for ambulation. The external fixator was removed after six weeks. At 12 weeks post-surgery, a pelvic X-ray showed a 12 mm separation of the symphysis (Fig. 1). Despite experiencing mild pain, the patient was able to walk without assistance. Four years later, she had an uneventful pregnancy and cesarean delivery. Fourteen years after surgery, she walked independently without pain. Her Lindahl score was 80 points (excellent).

### Case 2

A 25-year-old woman (gravida I, para I) was admitted to the department three months after an uneventful natural vaginal delivery. Since giving birth, the patient had been experiencing persistent discomfort in the region surrounding the pubic symphysis and iliosacral joints, significantly impacting her ability to walk. Standard treatments had proven unsatisfactory.

**Fig. 2:**
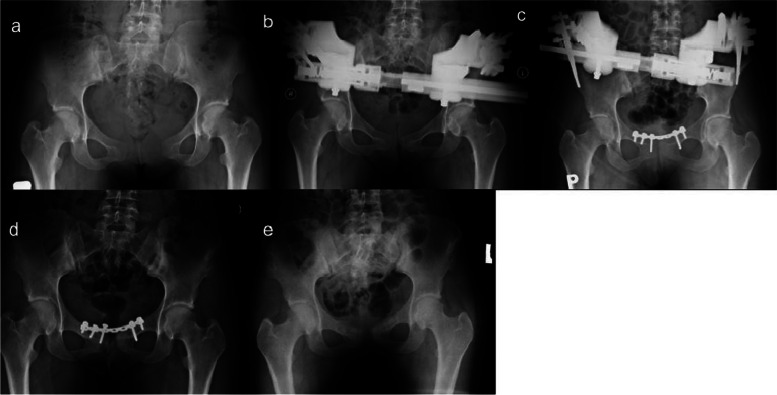
Case 2. A 25-year-old woman was admitted to the department 3 months after delivery. **a** Initial plain radiograph showing the pubic symphysis diastasis (APC II). **b** A radiograph was taken after performing closed reduction using an external fixator, resulting in a symphysis diastasis of 12 mm. **c** An additional internal fixation was performed using a 6-hole plate to reduce remaining diastasis. **d** Eleven-month follow-up radiograph shows a 12 mm gap in the reduction of the pubic symphysis. **e** One year after the removal of screws and a plate

An X-ray of the pelvis revealed a ruptured pubic symphysis with a 55 mm diastasis width and an 8 mm vertical gap. Additionally, there was an increased space in both iliosacral joints and osteosclerotic changes in the right joint (Fig. 2A), classifying it as an APC III pelvic injury. Closed reduction with external fixation using a Monotube fixator was initially performed, but the reduction of the pubic symphysis diastasis to only 15 mm was considered unsatisfactory (Fig. 2B). Twelve days later, the patient underwent open reduction and internal fixation with a 7-hole plate (Fig. 2C). The external fixator remained in place for four weeks before being removed. The patient was allowed to ambulate with crutches the day following the initial surgery. Complaints gradually decreased, and approximately twelve weeks postoperatively, she still reported slight pain. At the one-year follow-up after the initial surgery, X-rays revealed loose screws in the right pubic bone and a widening of the symphysis up to 12 mm (Fig. 2D). Consequently, the plate and screws were removed (Fig. 2E). Following this procedure, the patient was lost to follow-up.

### Case 3

**Fig. 3:**
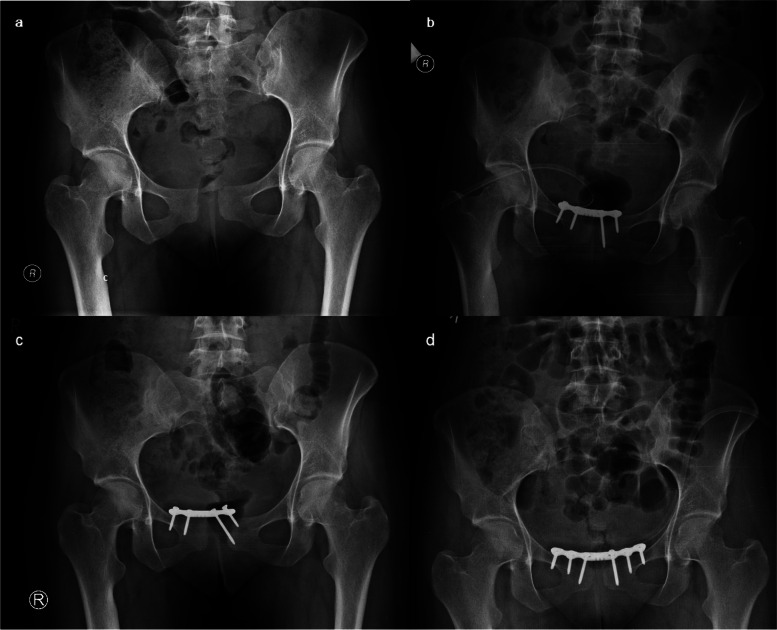
Case 3. Four months postpartum, a 30-year-old female was admitted to the department with pain in the pubic region. **a** Initial plain radiograph showing rupture of pubic symphysis with diastasis of 22 mm in width and 4 mm vertical. **b** The immediate postoperative film after open reduction and internal fixation of the pubic symphysis with a 4-hole plate. **c** Three-month follow-up radiograph showing plate failure. **d** The immediate postoperative film after revision surgery

A 30-year-old woman (gravida I, para I), four months post-childbirth, was admitted to the department due to discomfort in the pubic area. She had an uneventful vaginal delivery, and the newborn weighed 3950 g with a length of 54 cm. Around two months after giving birth, she began experiencing pain in the lower back region, particularly exacerbated during prolonged sitting. Relief was felt after resting, lying down, and at night, necessitating daily analgesic use. A pelvic x-ray revealed an APC II pelvic injury with a rupture of the pubic symphysis and a 22 mm width diastasis and 4 mm vertical displacement (Fig. 3A). The pubic symphysis was fixed with a 4-hole plate (Fig. 3B). The early postoperative course was uneventful. The patient was able to ambulate with crutches on the 1st postoperative day. Approximately two weeks post-discharge, discomfort in the pubic symphysis area recurred. Subsequent radiological imaging revealed loosening of screws and the plate after three months post-operation (Fig. 3C). The patient underwent reoperation, and re-reduction of the pubic symphysis with a dedicated reconstructive pelvic 6-hole plate was performed (Fig. 3D). The postoperative course was uneventful. Seven months after the revision surgery, the plate and screws were removed. Six years post-operation, the individual reported occasional soreness and distress localized to the pubic symphysis. These symptoms subsided following the rehabilitation process, and she moved independently without experiencing pain, with a Lindahl scale score of 75 points (good).

### Case 4

**Fig. 4:**
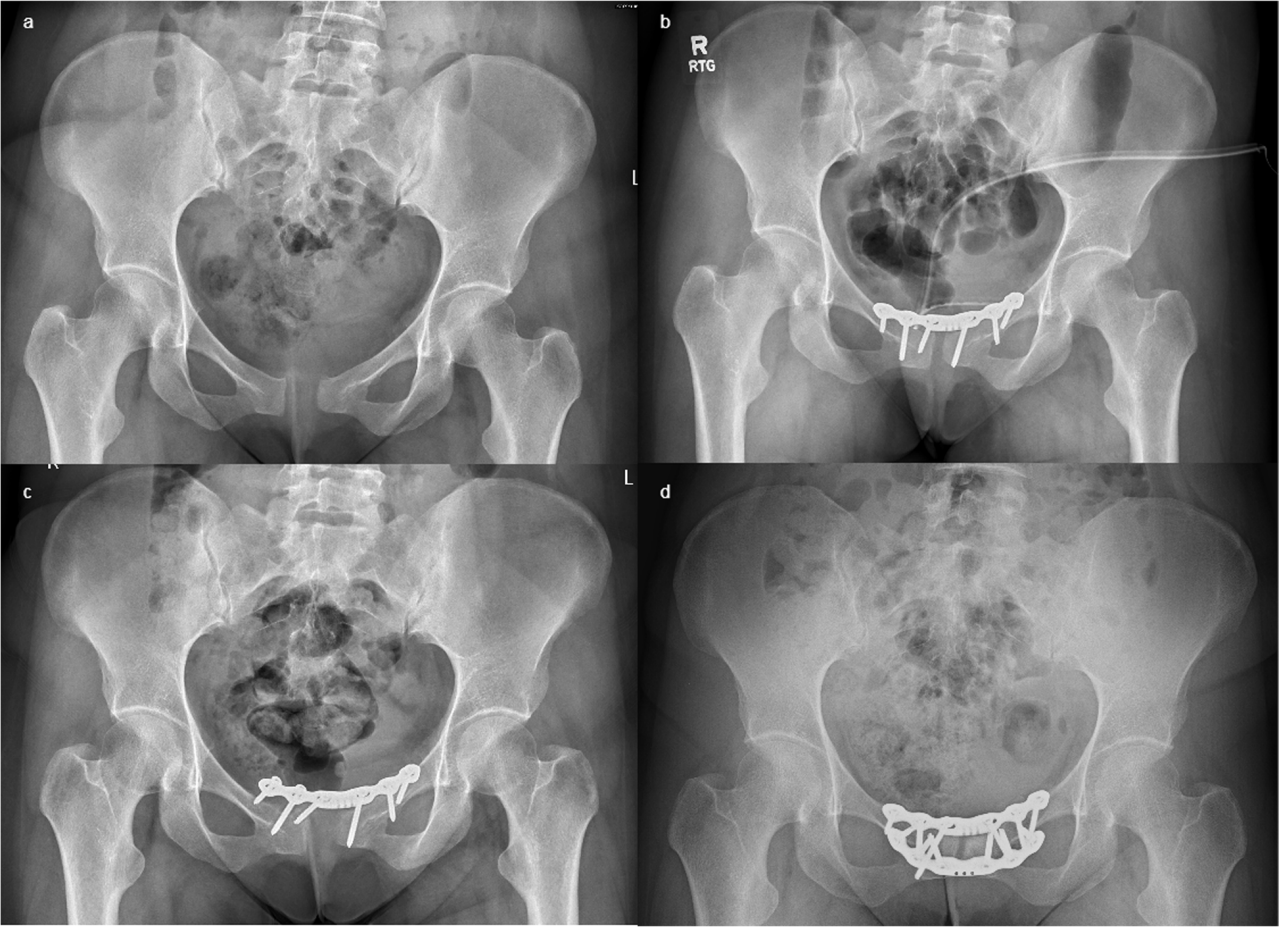
Case 4.A 30-year-old woman, who had given birth 6 months prior, complained of pain and a sensation of instability in her pelvis. **a** Initial plain radiograph shows a 20 mm wide and 6 mm vertical diastasis of the pubic symphysis. **b** The immediate postoperative film after open reduction and internal fixation of the pubic symphysis with a 6-hole plate. **c** Three-months follow-up radiograph showing plate failure. **d** Five-years follow-up radiograph after revision surgery, re-reduction and fixation with two orthogonal 6-hole plates. The pubic symphysis remains reduced

A 30-year-old woman (gravida I, para I) was admitted to our department 6 months after delivery, having undergone uneventful natural vaginal childbirth. The newborn’s birth weight was 3670 g, and its length was 57 cm. The patient reported enduring pain and an unsteady sensation in her pelvis since delivering her child. A radiograph revealed a dislocation of the pubic symphysis measuring 20 mm in width and 6 mm in vertical displacement (Fig. 4A). The diagnosis was a chronic symphysis rupture and APC II pelvic injury. Open reduction and internal fixation using a 6-hole pelvic plate were performed (Fig. 4B). The early postoperative course was uncomplicated, and the patient could walk with crutches on the 1st postoperative day. At the three-month follow-up visit, an X-ray revealed loose screws in the plate and a widened gap in the symphysis (Fig. 4C). After an additional 3 months, the pubic symphyseal dislocation was re-reduced and fixed with two 6-hole plates positioned orthogonally (Fig. 4D). The postoperative course was uneventful. The patient reported an absence of pain in the symphysis. After 5 years, the patient could walk independently without experiencing any pain. According to the Lindahl scale, her score was 73 (good).

### Case 5

**Fig. 5:**
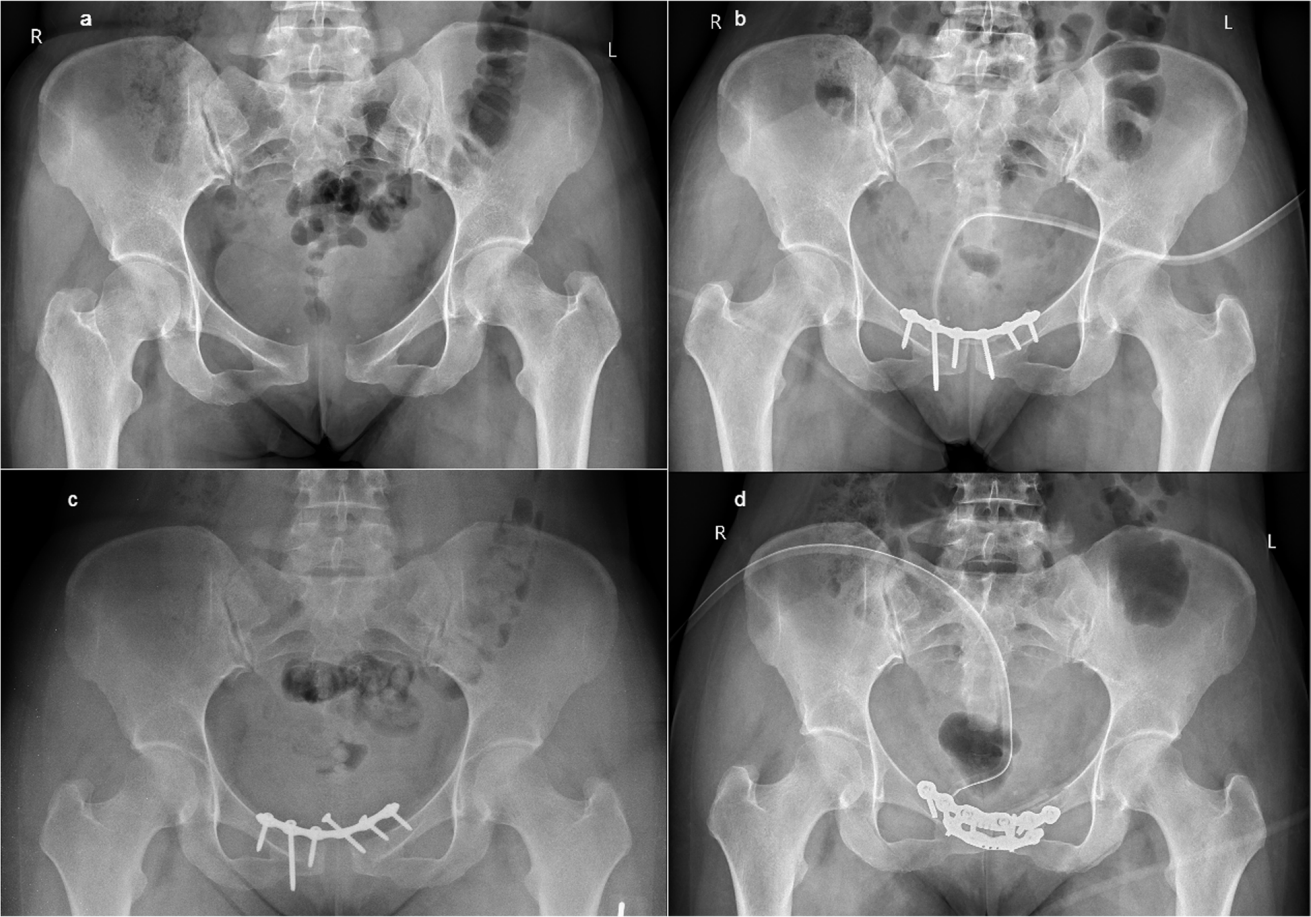
Case 5. A female aged thirty-eight was admitted to the hospital 1 year and 2 months after delivering her child, manifesting with discomfort around the pubic symphysis. **a** The initial plain radiograph shows a 21 mm wide and 2 mm vertical symphysis diastasis. **b** Open reduction internal fixation with a dedicated 6-hole plate. **c** Two months postoperatively, the plain radiograph shows plate failure. **d** The immediate postoperative film after revision surgery with fixation with two orthogonal 6 and 4-hole plates

A 38-year-old woman (gravida II, para II) was admitted to our department 14 months after her second natural vaginal delivery. The newborn weighed 3640 g at birth and measured 53 cm in length. Post-delivery, she developed pain over the pubic symphysis, and after a few weeks, pain also emerged in the iliosacral joints. Despite 14 months of conservative treatment without improvement, an AP radiograph revealed a 21 mm wide and 2 mm vertical diastasis of the symphysis, classified as an APC II injury (Fig. 5A). Open reduction and internal fixation of the pubic symphysis with a 6-hole symphysis plate were performed (Fig. 5B). The early postoperative course was uneventful, and the patient could ambulate with crutches on the 1st postoperative day. However, a follow-up x-ray 2 months after surgery indicated fixation failure and dislocation of the pubic symphysis (Fig. 5C). Subsequent revision surgery involved fixation with two symphysis plates with 6 and 4 holes (Fig. 5D). The early postoperative course was complication-free. The patient experiences periodic discomfort, but it does not impede everyday life. After 4.5 years, she walked independently without pain, and her Lindahl scale score was 78 points (excellent).

## Methods

The study protocol received approval from the Bioethics Committee at the Centre of Postgraduate Medical Education (17/2023 approved on 11.01.2023) and adhered to ethical standards outlined in the 2013 revision of the 1975 Declaration of Helsinki. The retrospective analysis utilized data from the Department of Pelvic Trauma and Pathology database at a tertiary care health center in Otwock, Poland, covering the period from January 2000 to December 2020.

During this period, 10 cases of pubic symphysis rupture during the peripartum period were identified. Among these, five patients were diagnosed with APC I and received conservative treatment, while the remaining five cases underwent surgical intervention. The decision for surgical treatment was based on criteria such as childbirth-onset pain, symphysis diastasis exceeding 10 mm and/or vertical instability greater than 5 mm, and non-responsiveness to conservative treatment in subacute or chronic stages. Surgical intervention involved open reduction and internal fixation of the symphysis pubis, performed using a standard Pfannenstiel surgical exposure.

Three patients were classified as APC II (equivalent to type B1), and two cases were classified as APC III (equivalent to type C) [12]. The average age of patients who underwent surgical treatment in our department was 31.4 years, ranging from 25 to 38 years. The average time from childbirth to surgery was 5.6 months, ranging from 1 to 14 months. One patient was lost for follow-up after 1 year. The mean follow-up was 7.4 years (range, 4.5-14). The functional outcome was evaluated using the Majeed score [13] modified by Lindahl [14]. All patients had improvements in symptoms. Good and excellent results were observed in all four patients available for follow-up, with a mean Lindahl score of 76.5 (range: 73-80).

### Literature data

**Fig. 6:**
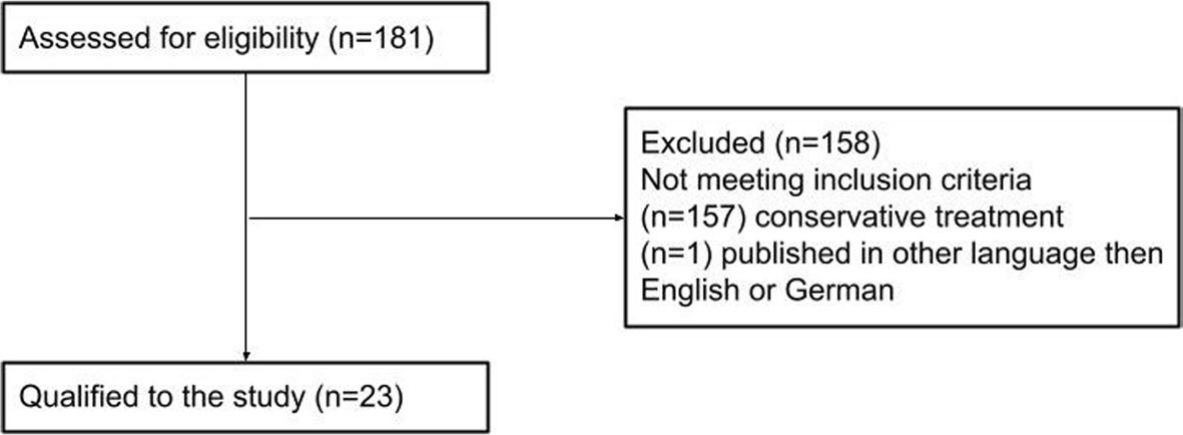
Flowchart of the literature review process

Furthermore, we conducted a literature review in the PubMed, Medline, and Cochrane databases, searching for entries related to symphysis AND disruption OR instability OR diastasis OR separation AND childbirth OR postpartum OR pregnancy OR peripartum OR delivery OR postnatal published between 1970 and December 2021. Two independent researchers conducted the review. We found 181 articles. Articles dealing with surgical treatment were included, with full text available and written in English or German. We found 23 reports [4, 7–10, 15–32] matching our inclusion and exclusion criteria with 37 patients described (Fig. 6). Five patients described in the current study were included, resulting in 42 patients.

Open reduction and internal fixation of the pubic symphysis were performed in 32 patients using a plate and screws. Additionally, nine patients underwent a closed reduction procedure with external fixation. In a single case, both fixation methods were utilized. Among these cases, seven involved additional fixation of the iliosacral joints: five with iliosacral screws, one with two plates, and one with a sacral bar (see Additional file 1).

Patients were divided into three groups based on the duration of symptoms since childbirth, following criteria from earlier publications [9, 33]: acute (up to 2 weeks) with 24 patients, subacute (2 weeks to 6 months) with 11 patients, and chronic (more than 6 months) with seven patients. The mean age was 30 years (range, 20-38) (Table 1).

**Table 1:** Data of patients who underwent surgery in the Department of Pelvic Trauma and Pathology

Case	Age	Duration of symptoms (months)	Duration of symptoms	Fixation	Complication	revision surgery	initial diastasis (mm)	final diastasis (mm)	follow-up (years)	Lindahl score (result, pts)
1	34	1	Subacute	ExFix	(−)	(−)	70	12	14	excellent, 80
2	25	3	Subacute	ExFix+Ant. Plate	screw loosening, partial loss of reduction	plate removal	55	10	N/A	N/A
3	30	4	Subacute	Ant. Plate	hardware failure, loss of reduction	larger ant. Plate	22	8	6	good, 75
4	30	6	Chronic	Ant. Plate	hardware failure, loss of reduction	two ant. Plates	20	5	5	good, 73
5	38	14	Chronic	Ant. Plate	hardware failure, loss of reduction	two ant. Plates	21	5	4.5	excellent, 78
	31.4	5.6					37.6	8	7.375	76.5

*ExFix *External fixator, *Ant. Plate* Anterior plate, *pts* points

In the acute group of 24 patients, 16 had the pubic symphysis fixed with a plate, and in 3 cases, the iliosacral joint was additionally fixed with screws. Complications related to fixing the anterior part of the pelvis were absent in this subgroup. However, there were instances of loosening of iliosacral screws, necessitating removal.

Eight cases in this group were treated with external fixators, with three cases (37.5%) experiencing complications (2 pin tract infections, one requiring revision surgery, and one loss of reduction). Overall, this group had 4 cases of complications (25%), and three patients (12,5%) underwent revision surgery.

In the subacute group (11 patients), 10 underwent open reduction and internal fixation (ORIF), 2 had external fixation (one with a dual fixation technique), and 2 requires additional iliosacral joint screw fixation (one with screws and one with plates). Complications occurred in six cases (54.5%), including a broken screw, five instances of screw loosening leading to four cases of loss of reduction, and one case of infection. Four of these complications require revision surgery, accounting for 36,3% of the cases (see Additional file 1).

The statistical analyses, conducted using the R statistical package (https://www.r-project.org/) revealed a higher number of complications in the subacute and chronic groups compared to the acute group (50% vs. 20%, *p* = 0.041). Additionally, within the acute group, more complications were observed in cases where external fixators were used (37.5% vs. 0%, *p* = 0.027). The results were considered significant with a *P* value of < 0.05.

## Discussion

We present a report on the surgical treatment of peripartum pubic symphysis rupture in five patients. In this cohort, four out of five women (75%) underwent revision surgery due to symphyseal construct failure. Postnatally, the symptoms manifested at an average of 6.75 months (ranging from 3 to 14 months).

During the one-year postoperative follow-up of the patient initially treated with external fixation (Case 2), who subsequently underwent plate fixation, it was observed that the screws had become loose, and the symphysis had recurrently widened, leading to the removal of the plate. The remaining three patients experienced failure of plate fixation, necessitating revision surgery at an average of 10.6 weeks (ranging from 6 to 12).

In this series, one patient (Case 3) underwent pubic symphysis fixation using a 4-hole plate, following the recommendation by Rommens et al. [8]. However, she experienced complete fixation failure, necessitating the removal of the initial fixation and subsequent fixation of the pubic symphysis using a 6-hole plate. Notably, Sagi and Papp’s [34] retrospective analysis suggests that the two-hole symphyseal plating technique group had a higher rate of implant failure and a significantly increased rate of pelvic malunion. Based on these findings, they recommend using multi-hole plating for unstable pubic symphyseal disruptions.

Two patients (Cases 4 and 5) underwent revision surgery with two orthogonal anterior plates. Ultimately, the distance between the pubic bones was 5-8 mm, and the Lindahl score assessment was determined to be good or excellent. It is noteworthy that the patient (Case 1), who initially had a 70 mm pubic symphysis diastasis, underwent external fixation 1 month after giving birth. This procedure was successful, although a 12 mm pubic symphysis diastasis remained.

It is essential to highlight that our analysis has revealed a notably higher rate of fixation failure than previously reported by Najibi et al. [9], who described 50% complications in subacute and chronic groups, and van Zwienen et al., who observed 47% complications within their cohort [35].

### Literature review

The literature review indicates that performing surgical treatment 2 weeks after delivery results in fewer complications compared to cases that are subacute or chronic (*P* = 0.041). Various factors may contribute to the clinical context. The pubic symphysis allows for small-magnitude movement of pubic bones during everyday activities. As humans ambulate, torsion of the sacrum (nutation) affects the front part of the pelvic ring [36, 37]. Walheim et al. found that the magnitude of anteroposterior sagittal movements when standing on alternate legs is 1.3 mm in nulliparous women and 2.1 mm in multiparous women [38]. Additionally, Garras et al. reported significant differences between the pelvic translations of nulliparous women (1.6 ± 0.8 mm) and multiparous women (3.1 ± 1.5 mm) [39].

In an early radiographic study from 1934, Abramson et al. [40] observed an average width of 7.7 mm at the symphysis in the last 2 months of pregnancy, indicating a mean increase of 3 mm compared to non-pregnant multiparous individuals serving as controls. The data mentioned suggest that the range of motion of pelvic joints during and after pregnancy increases. Therefore, if the patient begins walking before the pelvic ligaments have completely healed, it may lead to a failure of the construct [41, 42].

Previous biomechanical cadaveric studies have demonstrated that when symphyseal diastasis widens beyond 25 mm, the posterior iliosacral ligaments (sacrotuberous, sacrospinous, and the anterior iliosacral) become compromised in a sequential order [43]. This is classified as an APC II injury. In more severe cases, the posterior iliosacral ligaments may also be affected, resulting in a completely unstable APC III injury [2]. According to Matta [44], a fixation technique involving a single plate for pubic symphysis rupture is a reliable method. However, several subsequent studies have reported complications in treating APC II injuries with anterior fixation alone [41, 42].

Furthermore, Sagi et al. asserted that ligamentous damages to the iliosacral joint can be more substantial than what is seen on static imaging [45]. They conducted a stress examination under anesthesia with dynamic fluoroscopy, revealing occult instability of presumed APC I and APC II injuries. They concluded that inadequate treatment of wrongly identified trauma and chronic instability could contribute to unfavorable functional outcomes related to pelvic fractures.

Additionally, a retrospective study by Frank et al. revealed that using an anterior plate and an additional posterior screw for APC II pelvic ring injuries significantly reduces the incidence of anterior plate failure and malunion compared to using an anterior plate alone [46].

The ability of soft tissue to heal in the subacute and chronic group is poorer than that of the acute group, as mentioned by several authors [9, 35, 47]. When soft tissues such as ligaments, tendons, and muscles are torn, they often heal with contraction and shortening. Consequently, it can be challenging to reduce and maintain the reduction of chronic pelvic ring injuries [35, 39, 48]. Lybrand et al. [49] found that symphyseal cartilage removal in acute injury cases resulted in closer apposition of the pubic bones. This was associated with significantly reduced rates of implant failure and the need for revision surgery.

Najibi et al. suggested that the incapability to walk normally may lead to disuse osteopenia, making it difficult to achieve sound fixation [9]. Another specific factor for this group of patients is the excessive loss of bone during the third trimester of pregnancy, particularly during lactation [50, 51]. According to Athonvarangkul and Wysolmerski [52], a dramatic and reversible physiological response alters bone and mineral metabolism to accommodate the increased calcium demands for milk production throughout lactation. Research indicates that women may experience a decrease of up to 10% in their bone mineral content over 3-6 months of exclusive breastfeeding. However, full restoration of bone mineral content is achievable within 6-12 months of weaning [51, 52].

Our patients have reported difficulties in adhering to the postoperative weight-bearing protocol. As young mothers, they attended to their and their infants’ needs. Previous research has shown the challenge of enforcing partial weight-bearing restrictions on post-surgery patients. Vasarhelyi et al. demonstrated that most of their patients and healthy experimental controls exceeded their weight-bearing limitations following surgery [53]. Early loading increases the risk of implant breakdown since the osseous and soft tissue structures have insufficient time to heal.

Najibi et al. recommended using 6-hole plates and larger caliber screws (4.5 mm instead of 3.5 mm) to prevent failure in fixing the pubic symphysis. Other solutions, which include undertapping the screw tract [54, 55] or using K-wire pilot hole preparation, may improve screw pullout strength [56].

Furthermore, in subacute and chronic cases, fusing the symphysis was considered a better option than performing open reduction and internal fixation [9]. Weil et al. described a series of 19 patients who experienced persistent postpartum pelvic pain [57]. Most patients experienced pain relief with nonoperative treatment.

Nevertheless, four patients in this group underwent fusion of the symphysis pubis. In two cases, the iliosacral joint was also fixed with screws, and one patient had fusion of the iliosacral joint using 2 iliosacral screws. Three out of four patients experienced the resolution of their symptoms. One patient had symptoms of L5 nerve root irritation, and it was found that the iliosacral screw was malpositioned, necessitating its removal and the placement of a new screw. They recommended the use of percutaneous iliosacral fixation in cases where there are symptoms in the posterior area, corresponding changes in the X-rays, and a positive response to CT-guided injection into the iliosacral joint. Van Zwienen et al. demonstrated satisfactory results in severe pregnancy-related low back pain treated surgically using triple pelvic ring fixation, which includes symphysiodesis and bilateral percutaneous iliosacral screw fixation [35]. In the initial stage of the study, plates were used to fix the symphysis. However, after six cases of nonunion, a bone graft was added for further stabilization.

Two-plate fixation for peri-partum pubic symphysis rupture in acute cases with good outcomes was described by Hou et al. [4] and Yoo et al. [15]. Simonian et al. demonstrated on a cadaveric double-leg stance model that a “box plate fixation” using two-hole, 4.5 mm dynamic compression plates (DCP) positioned parallel to each other on the pubic symphysis results in the least amount of symphysis motion [58]. Yao et al. in their study using finite element analysis reported that the most effective pelvic fixation in both the anterior and posterior regions was achieved through the use of dual implants [59]. The data mentioned above, along with the positive mid-term results of the two revision cases described (Cases 4 and 5), indicate that utilizing two parallel plates could offer stable support for the pubic symphysis and serve as an alternative to symphysiodesis.

Analysis of literature cases indicates that the application of closed reduction and external fixation for acute cases is associated with more complications compared to anterior plate fixation. However, specific clinical scenarios may warrant the use of external fixators. For instance, Klotz et al. documented a case involving a complete longitudinal urethral rupture, pubic symphysis rupture, and pelvic fracture during spontaneous vaginal delivery [16]. Successful adaptation of the urethra was achieved after external skeletal fixation was applied to stabilize the pelvic fracture. Using an external fixator might be preferred over plate fixation in situations with a high risk of soft tissue infection.

Limited data exist regarding deliveries following the fixation of the pubic symphysis with a plate or symphysiodesis. We present the case of a patient (Case 1) who initially had the pubic symphysis fixed using an external fixator, which was later removed. Four years later, she underwent an uneventful pregnancy and cesarean delivery. According to Najibi et al., vaginal delivery after plate fixation is considered “not contraindicated but not recommended” [9]. However, symphysis fusion suggests a preference for cesarean section. Osterhoff et al. reported an incident-free vaginal delivery 15 months after the surgical fixation of postpartum symphyseal rupture [10].

Out of the five patients in our case series, only two (Cases 1 and 2) were referred to our department by obstetricians. The remaining three independently sought treatment, contributing to the delayed initiation of surgical intervention. This underscores the importance of a close collaboration between obstetricians and orthopedic surgeons. Recognizing the critical role of the time elapsed since delivery to surgery, early diagnosis by obstetricians can facilitate prompt intervention by the orthopedic team if required.

**Fig. 7:**
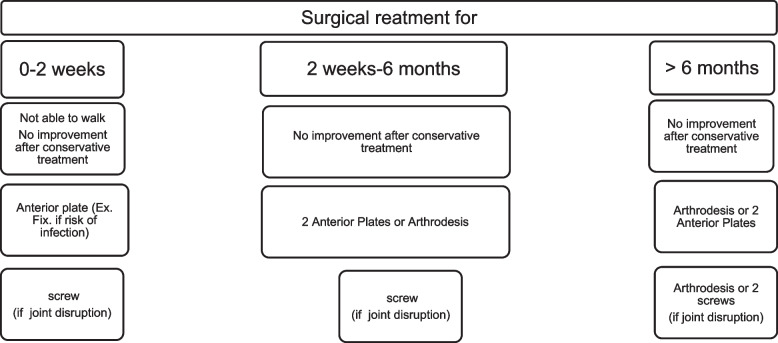
Proposed Algorithm for Treating Pubic Symphysis Rupture during the Peripartum Period based on Time Elapsed since Delivery

Several treatment algorithms have been proposed for acute peri-partum pubic symphysis rupture by Herren et al. [60] and Osterhoff et al. [10], as well as for chronic pelvic postpartum pain by Weil et al. [57]. We present an algorithm that takes into account the duration since childbirth (Fig. 7).

The study has some limitations. It was conducted retrospectively and lacked a control group. The primary constraint is the small sample size and the application of various treatment approaches to a limited cohort, possibly introducing bias. Given the rarity of this complication, assembling sizable and comparable patient groups is challenging. To provide a more comprehensive view, a literature review was conducted, resulting in a heterogeneous study group. Nonetheless, a protocol for managing such clinical conditions should be established as part of a prospective multicenter study.

## Conclusions

In our cohort, surgically treated pubic symphyseal dislocations showed increased complications when treated sub-acutely/chronically compared to acutely (50% vs. 20%, *p* = 0.041). Complications were higher in cases where external fixation was utilized than plate fixation (37.5% vs. 0%, *p* = 0.027). Surgical treatment performed more than 2 weeks after delivery is associated with a higher rate of complications. For patients with pain persisting longer than 2 weeks considering another vaginal delivery, orthogonal (90-90) plate fixation (“box plate fixation”) is recommended, and for patients who do not consider further vaginal parturition, symphysiodesis is recommended.

### Supplementary Information

**Additional file 1.** Overview of the research on surgical treatment for pubic symphysis rupture during childbirth [61–65].

## Data Availability

The datasets used and/or analyzed during the current study are available from the corresponding author upon reasonable request. Readers can access the data and material supporting the conclusions of the study by contacting.

## Abbreviations

APC: Anterior posterior compression
ORIF: Open reduction internal fixation
CREF: Closed reduction external fixation

## References

[1] Alicioglu B, Kartal O, Gurbuz H, Sut N (2008). Symphysis pubis distance in adults: a retrospective computed tomography study. Surg Radiol Anat.

[2] Wang Y, Li Y-Q, Tian M-R, Wang N, Zheng Z-C (2021). Role of relaxin in diastasis of the pubic symphysis peripartum. World J Clin Cases.

[3] Shnaekel KL, Magann EF, Ahmadi S (2015). Pubic Symphysis rupture and separation during pregnancy. Obstet Gynecol Surv.

[4] Hou Z, Riehl JT, Smith WR, Strohecker KA, Maloney PJ (2011). Severe postpartum disruption of the pelvic ring: report of two cases and review of the literature. Patient Saf Surg.

[5] Young JW, Burgess AR, Brumback RJ, Poka A (1986). Pelvic fractures: value of plain radiography in early assessment and management. Radiology.

[6] Tile M (1988). Pelvic ring fractures: should they be fixed?. J Bone Joint Surg (Br).

[7] Luger EJ, Arbel R, Dekel S (1995). Traumatic separation of the symphysis pubis during pregnancy: a case report. J Trauma.

[8] Rommens PM (1997). Internal fixation in postpartum symphysis pubis rupture: report of three cases. J Orthop Trauma.

[9] Najibi S, Tannast M, Klenck RE, Matta JM (2010). Internal fixation of symphyseal disruption resulting from childbirth. J Orthop Trauma.

[10] Osterhoff G, Ossendorf C, Ossendorf-Kimmich N, Zimmermann R, Wanner GA, Simmen H-P, Werner CML (2012). Surgical stabilization of postpartum symphyseal instability: two cases and a review of the literature. Gynecol Obstet Investig.

[11] Kharrazi FD, Rodgers WB, Kennedy JG, Lhowe DW (1997). Parturition-induced pelvic dislocation: a report of four cases. J Orthop Trauma.

[12] Burgess AR, Eastridge BJ, Young JW, Ellison TS, Ellison PS, Poka A, Bathon GH, Brumback RJ (1990). Pelvic ring disruptions: effective classification system and treatment protocols. J Trauma.

[13] Majeed SA (1989). Grading the outcome of pelvic fractures. J Bone Joint Surg (Br).

[14] Lindahl J, Hirvensalo E, Böstman O, Santavirta S (1999). Failure of reduction with an external fixator in the management of injuries of the pelvic ring. Long-term evaluation of 110 patients. J Bone Joint Surg (Br).

[15] Yoo JJ, Ha Y-C, Lee Y-K, Hong JS, Kang B-J, Koo K-H (2014). Incidence and risk factors of symptomatic peripartum diastasis of pubic symphysis. J Korean Med Sci.

[16] Klotz T, Derakhshani P, Vorreuther R, Engelmann U (1998). Complete urethral rupture with symphysis injury and anterior pelvic ring fracture during spontaneous delivery. Urologe A.

[17] Erickson D, Low J, Shumway J (2016). Management of Postpartum Diastasis of the pubic Symphysis. Orthopedics.

[18] Pennig D, Gladbach B, Majchrowski W (1997). Disruption of the pelvic ring during spontaneous childbirth. A case report. J Bone Joint Surg (Br).

[19] Hierholzer C, Ali A, Toro-Arbelaez JB, Suk M, Helfet DL (2007). Traumatic disruption of pubis symphysis with accompanying posterior pelvic injury after natural childbirth. Am J Orthop.

[20] Petersen AC, Rasmussen KL (1992). External skeletal fixation as treatment for total puerperal rupture of the pubic symphysis. Acta Obstet Gynecol Scand.

[21] Shuler TE, Gruen GS (1996). Chronic postpartum pelvic pain treated by surgical stabilization. Orthopedics.

[22] Kotwal PP, Mittal R (1998). Disruption of the symphysis pubis during vaginal delivery. A case report. J Bone Joint Surg Am.

[23] Heath T, Gherman RB (1999). Symphyseal separation, sacroiliac joint dislocation and transient lateral femoral cutaneous neuropathy associated with McRoberts’ maneuver. A case report. J Reprod Med.

[24] Seth S, Das B, Salhan S (2003). A severe case of pubic symphysis diastasis in pregnancy. Eur J Obstet Gynecol Reprod Biol.

[25] Chang D, Markman BS (2002). Images in clinical medicine. Spontaneous resolution of a pubic-symphysis diastasis. N Engl J Med.

[26] Shippey S, Roth J, Gaines R (2013). Pubic symphysis diastasis with urinary incontinence: collaborative surgical management. Int Urogynecol J.

[27] Gräf C, Sellei RM, Schrading S, Bauerschlag DO (2014). Treatment of parturition-induced rupture of pubic symphysis after spontaneous vaginal delivery. Case Rep Obstet Gynecol.

[28] Pires R, Labronici PJ, Giordano V, Kojima KE, Kfuri M, Barbisan M, Wajnsztejn A, de Andrade M (2015). Intrapartum pubic Symphysis disruption. Ann Med Health Sci Res.

[29] Buitendyk M, Brennan B, Vora P, Smith P, Winsor S (2018). Acute Intrapartum rupture of the pubic Symphysis requiring resuscitation and surgical intervention: a case report. J Obstet Gynaecol Can.

[30] Norvilaite K, Kezeviciute M, Ramasauskaite D, Arlauskiene A, Bartkeviciene D, Uvarovas V (2020). Postpartum pubic symphysis diastasis-conservative and surgical treatment methods, incidence of complications: two case reports and a review of the literature. World J Clin Cases.

[31] Müller M, Greve F, Zyskowski M, Wurm M, Biberthaler P, Kirchhoff C (2021). External fixation for treatment of peripartum pubic symphysis separation : clinical case and discussion. Unfallchirurg.

[32] Dunivan GC, Hickman AM, Connolly A. Severe separation of the pubic symphysis and prompt orthopedic surgical intervention. Obstet Gynecol. 2009;114:473–5. 10.1097/AOG.0b013e3181998bd1 19622966

[33] Kovacs FM, Abraira V, Zamora J, Fernández C, Spanish Back Pain Research Network (2005). The transition from acute to subacute and chronic low back pain: a study based on determinants of quality of life and prediction of chronic disability. Spine.

[34] Sagi HC, Papp S (2008). Comparative radiographic and clinical outcome of two-hole and multi-hole symphyseal plating. J Orthop Trauma.

[35] van Zwienen CMA, van den Bosch EW, Snijders CJ, van Vugt AB (2004). Triple pelvic ring fixation in patients with severe pregnancy-related low back and pelvic pain. Spine.

[36] Lavignolle B, Vital JM, Senegas J, et al. An approach to the functional anatomy of the sacroiliac joints in vivo. Anat Clin. 1983;5(3):169–76. 10.1007/BF01799002 6671062

[37] Becker I, Woodley SJ, Stringer MD. The adult human pubic symphysis: a systematic review. J Anat. 2010;217(5):475–87. 10.1111/j.1469-7580.2010.01300.x. 10.1111/j.1469-7580.2010.01300.x PMC3035856 20840351

[38] Walheim G, Olerud S, Ribbe T. Mobility of the pubic symphysis. Measurements by an electromechanical method. Acta Orthop Scand. 1984. 10.3109/17453678408992338 6545936

[39] Garras DN, Carothers JT, Olson SA (2008). Single-leg-stance (flamingo) radiographs to assess pelvic instability: how much motion is normal?. J Bone Joint Surg Am.

[40] Abramson D (1934). Relaxation of the pelvic joints in pregnancy. Surg Gynecol Obstet.

[41] Moed BR, Grimshaw CS, Segina DN. Failure of locked design-specific plate fixation of the pubic symphysis. J Orthop Trauma. 2012;26(7):e71–5. 10.1097/BOT.0b013e31822c8396. 10.1097/BOT.0b013e31822c8396 22183198

[42] Eastman JG, Krieg JC, Routt MLC. Early failure of symphysis pubis plating. Injury. 2016;47(8):1707–12. 10.1016/j.injury.2016.05.019. 10.1016/j.injury.2016.05.019 27282685

[43] Vrahas M, Hern TC, Diangelo D, Kellam J, Tile M. Ligamentous contributions to pelvic stability. Orthopedics. 1995;18(3):271–4. 10.3928/0147-7447-19950301-09. 10.3928/0147-7447-19950301-09. 10.3928/0147-7447-19950301-09 7761317

[44] Matta JM, Saucedo T. Internal fixation of pelvic ring fractures. Clin Orthop Relat Res. 1989;(242):83–97. 2706863

[45] Sagi HC, Coniglione FM, Stanford JH. Examination under anesthetic for occult pelvic ring instability. J Orthop Trauma. 2011;25(9):529–36. 10.1097/BOT.0b013e31822b02ae. 10.1097/BOT.0b013e31822b02ae 21857421

[46] Avilucea FR, Whiting PS, Mir H. Posterior Fixation of APC-2 Pelvic Ring Injuries Decreases Rates of Anterior Plate Failure and Malunion. J Bone Joint Surg Am. 2016;98(11):944–51. 10.2106/JBJS.15.00723. 10.2106/JBJS.15.00723 27252439

[47] Grace JN, Sim FH, Shives TC, Coventry MB (1989). Wedge resection of the symphysis pubis for the treatment of osteitis pubis. J Bone Joint Surg Am.

[48] Hagen R (1974). Pelvic girdle relaxation from an orthopaedic point of view. Acta Orthop Scand.

[49] Lybrand K, Kurylo J, Gross J, Templeman D, Tornetta P (2015). Does removal of the Symphyseal cartilage in Symphyseal dislocations have any effect on final alignment and implant failure?. J Orthop Trauma.

[50] Wysolmerski JJ (2010). Interactions between breast, bone, and brain regulate mineral and skeletal metabolism during lactation. Ann N Y Acad Sci.

[51] Telfer S, Brunnquell CL, Allen JD, Linnau KF, Zamora D, Kleweno CP (2021). The effect of age and sex on pelvic bone density measured opportunistically in clinical CT scans. J Orthop Res.

[52] Crosstalk within a brain-breast-bone axis regulates mineral and skeletal metabolism during lactation. Front Physiol. 2023;14. 10.3389/fphys.2023.1121579. 10.3389/fphys.2023.1121579 PMC9979219 36875035

[53] Vasarhelyi A, Baumert T, Fritsch C, Hopfenmüller W, Gradl G, Mittlmeier T. Partial weight bearing after surgery for fractures of the lower extremity–is it achievable? Gait Posture. 2006;23(1):99–105. 10.1016/j.gaitpost.2004.12.005 16311201

[54] Kuklo TR, Lehman RA (2003). Effect of various tapping diameters on insertion of thoracic pedicle screws: a biomechanical analysis. Spine.

[55] Lehman RA, Kang DG, Wagner SC (2015). Management of osteoporosis in spine surgery. J Am Acad Orthop Surg.

[56] Basmajian HG, Liu JN, Scudday T, Campbell ST, Amin NH (2020). Kirschner wire prepared pilot holes improve screw pullout strength in synthetic osteoporotic-type bone. J Clin Orthop Trauma.

[57] Weil YA, Hierholzer C, Sama D, Wright C, Nousiainen MT, Kloen P, Helfet DL (2008). Management of persistent postpartum pelvic pain. Am J Orthop.

[58] Simonian PT, Routt ML, Harrington RM, Tencer AF (1994). Box plate fixation of the symphysis pubis: biomechanical evaluation of a new technique. J Orthop Trauma.

[59] Yao F, He Y, Qian H, Zhou D, Li Q (2015). Comparison of biomechanical characteristics and pelvic ring stability using different fixation methods to treat pubic Symphysis diastasis: a finite element study. Medicine.

[60] Herren C, Sobottke R, Dadgar A, Ringe MJ, Graf M, Keller K, Eysel P, Mallmann P, Siewe J (2015). Peripartum pubic symphysis separation--Current strategies in diagnosis and therapy and presentation of two cases. Injury.

[61] Dunivan GC, Hickman AM, Connolly A (2009). Severe separation of the pubic symphysis and prompt orthopedic surgical intervention. Obstet Gynecol.

[62] Kellam JF. The role of external fixation in pelvic disruptions. Clin Orthop Relat Res. 1989:66–82. 2647337

[63] Pohlemann T, Bosch U, Gänsslen A, Tscherne H. The Hannover experience in management of pelvic fractures. Clin Orthop Relat Res. 1994:69–80. 8050249

[64] Williams PR, Thomas DP, Downes EM (2000). Osteitis pubis and instability of the pubic symphysis. When nonoperative measures fail. Am J Sports Med.

[65] Mobility of the pubic symphysis: Measurements by an electromechanical method. Acta Orthopaedica Scandinavica. 1984;55(2):203–8. 10.3109/17453678408992338. 10.3109/17453678408992338 6545936

